# Improving the dictionary lookup approach for disease normalization using enhanced dictionary and query expansion

**DOI:** 10.1093/database/baw112

**Published:** 2016-08-08

**Authors:** Jitendra Jonnagaddala, Toni Rose Jue, Nai-Wen Chang, Hong-Jie Dai

**Affiliations:** ^1^School of Public Health and Community Medicine, UNSW, Kensington, NSW 2033, Australia; ^2^Prince of Wales Clinical School, UNSW, Kensington, NSW 2033, Australia; ^3^Institution of Information Science, Academia Sinica, Taipei 115, Taiwan; ^4^Graduate Institute of Biomedical Electronics and Bioinformatics, National Taiwan University, Taipei, Taiwan and; ^5^Department of Computer Science and Information Engineering, National Taitung University, Taipei, Taiwan

## Abstract

The rapidly increasing biomedical literature calls for the need of an automatic approach in the recognition and normalization of disease mentions in order to increase the precision and effectivity of disease based information retrieval. A variety of methods have been proposed to deal with the problem of disease named entity recognition and normalization. Among all the proposed methods, conditional random fields (CRFs) and dictionary lookup method are widely used for named entity recognition and normalization respectively. We herein developed a CRF-based model to allow automated recognition of disease mentions, and studied the effect of various techniques in improving the normalization results based on the dictionary lookup approach. The dataset from the BioCreative V CDR track was used to report the performance of the developed normalization methods and compare with other existing dictionary lookup based normalization methods. The best configuration achieved an F-measure of 0.77 for the disease normalization, which outperformed the best dictionary lookup based baseline method studied in this work by an F-measure of 0.13.

**Database URL:** https://github.com/TCRNBioinformatics/DiseaseExtract

## Introduction

**Figure 1.**
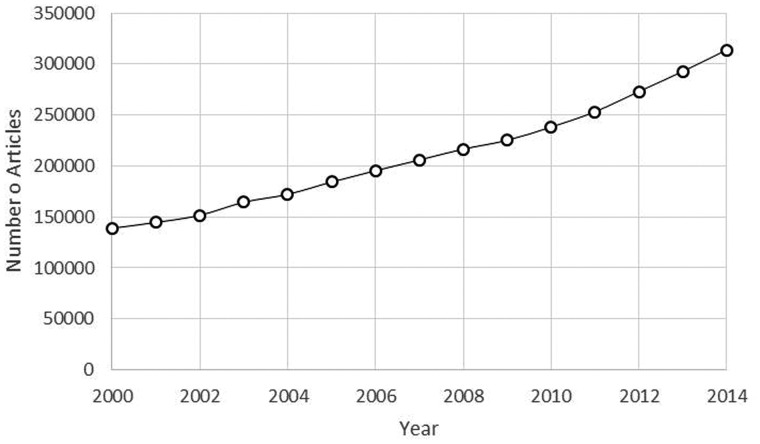
Number of publications resulting from the search query ‘disease OR diseases OR disorder OR disorders’ from 2000 to 2014.

The importance of extracting disease related information mapped to a standardized vocabulary is increasing with the yearly increase of published biomedical literature (1). It is revealed that in 2011, over 20 million documents were available in PubMed alone with an average of 4% increase per year with keywords relating to diseases being the second most common user search query (1). A PubMed query using the keywords ‘disease OR diseases OR disorder OR disorders’ in early 2016 resulted in over 6.5 million documents revealing an average of 6% yearly increase from 2000 to 2014 (Figure 1). Comparable trends can also be observed in specific disease categories such as cancer and cardio vascular diseases. Because of this increase in available literature, researchers are now faced with the challenge of identifying biomedical documents relevant to them (2,3). Medical subject headings (MeSH) terminology was developed by the National Library of Medicine to speed up and increase the precision of biomedical literature retrieval (4). Where possible, documents in PubMed are indexed with relevant disease specific keywords using MeSH terminology. Manually assigning disease specific MeSH terms to documents is a labor- and time-intensive process which would require monetary investment as well. Text mining techniques can be employed to assist in overcoming these challenges (5).

Over the years, various methods have been proposed to extract disease related information (6–9). Typically, these methods include two broad stages: (i) extracting disease mentions from free text–a task referred to as disease named entity recognition (DNER) and (ii) normalizing the recognized mentions to standard controlled vocabularies such as MeSH–a task referred to as disease name normalization (DNORM). However, performing DNER and DNORM is not straightforward and often complex (7, 10). Past issues that have been observed include limitations in the size and annotation of the corpora used for developing DNER and DNORM systems (9, 11). Disease mentions are also observed to be highly ambiguous with varying terms and definitions. Additionally, abbreviations are commonly used to represent disease in literature. This poses a problem since one abbreviation may represent multiple terms (11).

Among all of the proposed approaches, a probabilistic method known as conditional random fields (CRFs) is widely used for DNER (12–17). Similarly, dictionary lookup method is for DNORM (11, 18, 19). The dictionary lookup method relies heavily on string matching, which can either be exact or partial matching (i.e. proximity and fuzzy matching). The dictionary lookup approach has various advantages and is known to provide competitive results because the usage of disease terminology is standardized in biomedical literature (12, 19). The major advantage of this method is its scalability. When compared to machine learning based DNORM, dictionary lookup is fast and can be easily scaled to very large collections of free text documents such as PubMed snapshots (14). In addition, unlike machine learning methods such as learning to rank for normalization, the dictionary lookup method does not require training data which typically is annotated for a specific purpose and domain such as gene normalization. Dictionary lookup method which requires standard dictionaries is more generic and can be easily ported to other domains by simply switching the dictionaries. Although the dictionary lookup approach is not effective in handling domain specific variations, Shah *et al.* (20) demonstrated that the approach could achieve competetive results in recognizing biomedical concepts when used with the right combination of additional techniques including abbreviation resolution, enhanced dictionary (21), query expansion (22, 23) and priority rules. The combined effects of these techniques on dictionary-based DNORM are not well explored previously. We are only aware of Kang *et al.* (24) who explored the impact of using rules based on linguistic information like shallow parsing and part of speech tags. Additionally, as per our knowledge, no previous study has compared various dictionary lookup methods for DNORM. In light of this, we explore the impact of introducing additional techniques on the dictionary lookup based DNORM. After comparing to other similar dictionary-based methods, our results suggest that, with the right combination of additional techniques we can significantly improve the performance of the dictionary lookup based DNORM.

## Related work

Broadly, the usage of dictionary lookup in disease identification tools and studies can be classified into two types: (i) Both DNER and DNORM are performed using dictionary lookup; and (ii) Just DNORM is performed using dictionary lookup. In the first type, the mentions (single terms or phrases) from standard vocabularies are matched against free text, often subjected to a few additional steps like pre-processing and query expansion. In other words, both DNER and DNORM happen at the same time. In the second type, DNER is initially performed using machine learning based approaches which is followed by the dictionary lookup based normalization.

Clinical Text Analysis and Knowledge Extraction System (cTAKES), a modular system based on Unstructured Information Management Architecture framework (UIMA) and OpenNLP package is a good example for the first type of disease identification tools (25). cTAKES enhances its dictionaries by adding synonyms from UMLS and additional custom entries maintained by Mayo clinic. YTEX (26) improved cTAKES dictionary lookup by performing word sense disambiguation using semantic similarities calculated using the adapted Lesk algorithm. MetaMap is another such tool which finds noun phrases in the text first and then performs dictionary lookup after (18). MetaMap represents strings of the noun phrase as queries and expands the queries by generating lexical variants (not limited to spelling, inflection and punctuation variants). For example, ‘anaesthetic’ and ‘anesthetic’ are spelling variants. MetaMap then disambiguates concepts based on a custom score. Open Biomedical Annotator (OBA) web service implements radix-tree-based data structure to extract disease information from text by performing dictionary lookup using ontologies (20, 27). The dictionaries used for lookup in OBA are built by pooling concepts from ontologies. In addition to dictionary lookup, OBA performs semantic expansion to identify final concepts by leveraging the hierarchical and mapping information of ontologies. BeCAS is another web service to annotate diseases and several other entity types (28). BeCAS uses deterministic finite automatons for dictionary lookup. MetMap, OBA and BeCAS tools do not perform any dictionary enhancements. Unlike them, cTAKES generates non-lexical variants (variations of head and modifiers within noun phrases) and YTEX enriches dictionaries by adding lexical variants. Almost all the dictionary lookup based tools perform some sort of query expansion. In terms of abbreviation resolution, to the best our knowledge, cTAKES does not have any abbreviation resolution. BeCAS, MetaMap and OBA are capable of resolving abbreviations by query expansion. Please refer to Appendix 1 for more detailed comparison of the related tools discussed.

Often researchers used tools like MetaMap, cTAKES, BeCAS, OBA and YTEX in combination. Khare *et al.* (29) used MetaMap to recognize and normalize diseases in DailyMed drug database. Shah *et al.* (20) used it to compare MetaMap’s disease DNER performance with Mgrep (30). Patrick *et al.* (31, 32) used CRFs and support vector machines (SVM) to perform DNER followed by a dictionary lookup based DNORM in conjunction with a few rules in clinical notes. Zuccan *et al.* (15) also used CRFs to perform DNER followed by DNORM using MetaMap. Xia *et al.* (33) used both MetaMap and cTAKES together to perform DNER and DNORM. They merged output from both tools and resolved conflicts using a simple algorithm.

## Methods

**Figure 2.**
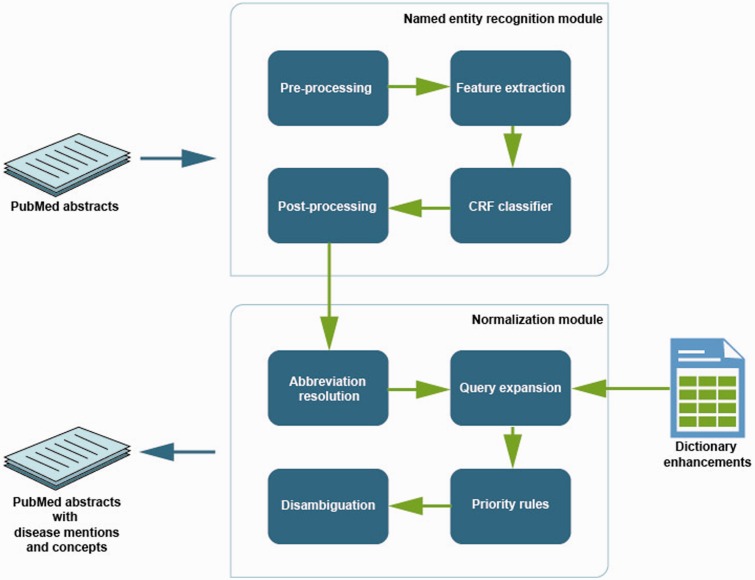
Overview of methods to extract disease information from the text.

The overview of the proposed methods is illustrated in Figure 2. The methods in this study are an extension to our previous work as part of the BioCreative V challenge (34, 35). The named entity recognition (NER) and normalization modules are the two main components. Initially, the documents were pre-processed using the Stanford PTBTokenizer (http://nlp.stanford.edu/software/tokenizer.shtml). The modules are explained more in detail in the following sections.

### Disease named entity recognition

The DNER task is formulated as a sequential labeling problem using CRFs. CRFs outperformed both supervised and unsupervised approaches in various tasks such as entity recognition, speech recognition and machine translation (7, 36, 37). Thus, we choose CRFs for implementing our DNER module. In short, CRFs are a probabilistic framework for performing sequential labeling task. Contrary to the classification algorithms like SVMs, CRFs are contextual because of their Markov property. Forward backward and Viterbi are the most commonly used algorithms to infer sequence in CRFs. In this study, first order CRFs with Viterbi algorithm for inference and Quasi-Newton optimization from Stanford’s CRF-NER package (38) were used.

**Figure 3.**
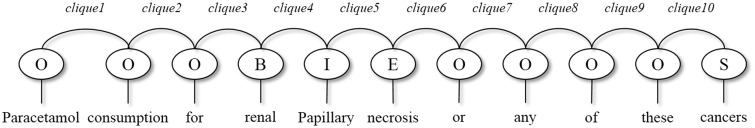
Example of BIESO tagging format used in this study and graphical representation of ‘paracetamol consumption for renal papillary necrosis or any of these cancers’ tagged as [O, O, O, B, I, E, O, O, O, O, S].

Given an input sequence of tokens *W*, a linear-chain CRF (Equation 1) computes the conditional probability associated with its corresponding hidden labeled sequence *Y*; where Z(W) is the normalization factor that makes the probability of all state sequences sum to one; C is the set of all cliques in this sentence; and c is a single clique, which reflects the position of the current word, as displayed in Figure 3. The function $f_{i}(Y_{c-1,\;}Y_{c,}\;W,c)$ is a binary-valued feature function whose learned weight is$\;\lambda_{i}$. Large positive values of $\lambda_{i}$ indicate a preference for such the corresponding feature.

(1) $${\text{p}}_{}\left(Y|W\right)=\frac{1}{Z\left(W\right)}\exp\left(\sum_{c\in C}\sum_{i}\lambda_{i}f_{i}\left(Y_{c-1},\;Y_{c},W,c\right)\right)$$

In order to perform sequential labeling using CRFs, it is essential to represent the input token sets with a suitable tag set. BIO (B = beginning of an entity, I = inside an entity and O = outside of an entity) format is the most widely used tagging in sequential labeling. In this study, we employed BIESO (B-beginning, I-intermediate, E-end, S-single word entity and O-outside) tagging as it has been reported to perform better than BIO tagging (39–41). Figure 3 shows a sample sentence tagged using BIESO format. In this sample, there are two disease entities; one multi token entity—‘renal papillary necrosis’ and one single token disease entity ‘cancers’.

*Feature extraction*. The features were extracted using the Stanford CRF-NER (http://nlp.stanford.edu/software/CRF-NER.html) package. Features with weight (absolute value) below the threshold of 0.05 were dropped to avoid noisy features. We employed basic features that included word, gazetteer, morphological and word shape features. The features were then conjugated to obtain a new set of features. The context information can help CRFs predict the label of current token. Thus, we considered previous token, current token and next token by themselves and their part of speech information as word features. MEDIC vocabulary (42) was used to extract gazetteer features. We tokenized MEDIC vocabulary primary names and synonyms and tagged them using BIESO format. Two features were extracted depending on the match between token and entries in the gazetteer. One feature represented the length of the matched entry and the other feature represented presence of token match with an entry in the gazetteer. Identifying lemma and affixes of a word token helps in better understanding the underlying morpheme. We included lemma of previous, current and next tokens as morphological features. Similarly, we also included prefixes and suffixes (of length two to five characters) of previous, current and next tokens as morphological features.

Disease names in general are noun phrases and thus are represented with appropriate writing styles in literature. At the same time, diseases are often written in short forms. Word shape features can be used to capture the different writing styles by employing pattern matching using regular expressions (43, 44). Word shape features are orthographic features with more granularity when compared to traditional orthographic features. They encode structure of a word using simple representations. They can also be used to capture internal punctuation and Greek letters. For example, ‘CANCER’ would become ‘XXXXXX’ and whereas ‘Diabetes’ would become ‘Xxxxxxx’. The capital letters are represented by ‘X’, lowercase letters are represented by ‘x’ and similarly digits with ‘d’ and Greek letters with ‘g’. We identified numbers, punctuations, and words in lowercase, uppercase and capitalized patterns. We extracted the word shape features for current, next and previous tokens. In situations where the word shape features were not identified, by default they were assigned as ‘none’.

*Post-processing.* Before passing the recognized disease mentions to the normalization module, we took all the recognized entities (including abbreviations) and quickly searched the documents to check whether there were any entities which were recognized by the NER module in one situation or context but not recognized in the other. The search was based on exact string matching. A few studies have reported success in improving recall of CRF-based entity recognition with this type of post-processing step because the model predict labels based on local information only (45–47).

### Disease name normalization

MEDIC vocabulary was used to map the recognized disease mentions to MeSH concepts (42). The MEDIC vocabulary includes both MeSH and OMIM terminologies. In this study, we focused on the MeSH IDs and excluded the OMIM concepts for normalization. We formalized the normalization problem as following. Let Q = {*q*_1_, *q*_2_, … , *q*_n_} be an entity recognized by our DNER module with *n* terms and D = {*d*_1_, *d*_2_, … , *d*_n_} be an entry with *n* terms in the MEDIC vocabulary. Instead of retrieving top *k*, where *k* = {1, 2, …, *n*} entries for a given Q from the MEDIC vocabulary based on relevance calculated using function like *Score*(*Q*, *D*); which is more common; we retrieved entries that satisfy the condition Q = D.

**Figure 4.**
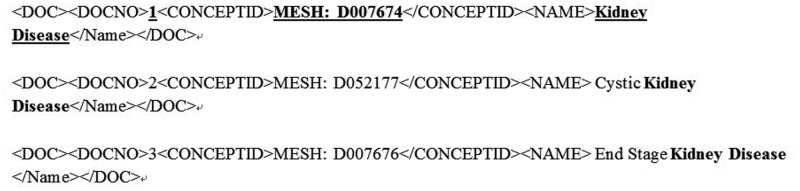
Sample dictionary entries.

In other words, the recognized disease mentions were checked against the MEDIC vocabulary for an exact string match. An exact match here is a string match where the words, number and order of words is exactly the same as an entry in the MEDIC vocabulary. Before performing the match, both Q and D were pre-processed to convert all terms into lower case, and punctuations and stop words were removed. For example, assume that the DNER module recognizes ‘Kidney Disease’ in the text and now consider the potential entries from MEDIC vocabulary for a match in Figure 4. Our method chooses the first entry (DOCNO: 1) and MeSH concept D007674 was returned.

*Abbreviation resolution*. A rule-based pattern matching abbreviation finder component was developed to identify abbreviations and their corresponding long forms in the text. The component used a custom lexicon of abbreviations which was developed from the training and development sets of the BioCreative V CDR dataset and the MEDIC vocabulary using BioText tool (48). The abbreviations lexicon included both short, long forms and occurrence frequency. Once our DNER module recognized disease mentions in an article, they were processed through abbreviations finder component for abbreviations. The identified abbreviations were checked against the lexicon and replaced with their long forms. For example, our DNER recognized two entities—‘myocardial infarction’ and ‘MI’ from an abstract with a sentence ‘To evaluate the safety of lidocaine in the setting of cocaine-induced myocardial infarction (MI)’. In our lexicon MI refers to ‘myocardial infarction’. Thus, we replaced MI with ‘myocardial infarction’. In situations where there were multiple long forms associated with a single short form; we checked whether one of those long forms were mentioned before in the document. If it was, then the short form was expanded with that particular long form. If there was no long form mentioned previously in the abstract, we assign the long form which had highest occurrence observed during development of abbreviation lexicon. If there was no long form found in lexicon, the recognized entity was left unexpanded.

*Enhanced dictionary and query expansion*. The MEDIC vocabulary includes valuable information such as primary names, synonyms, MeSH hierarchical details, definitions and broad groups. However, from our initial experiments we noticed that few disease mentions were expressed in fairly simple language without much medical jargon. These synonyms are not observed in MEDIC vocabulary but in WordNet (https://wordnet.princeton.edu/), which is not specific to a biomedical domain rather a generic synonyms database for the English language. For example, ‘Kidney Diseases’ in MEDIC do not include synonyms ‘renal disorder’ and ‘nephropathy’, which are found in WordNet for the same ‘kidney diseases’ phrase. The coverage of synonyms in MEDIC can be enhanced by adding missing synonyms from WordNet. Thus, every primary name phrase in MEDIC was checked for WordNet synonyms and added back to MEDIC, if they do not exist. Overall, 2036 WordNet synonym entries were added to 11 343 existing MEDIC primary names. There were 63 728 synonyms for MeSH concepts in the MEDIC vocabulary. Finally, the MEDIC vocabulary is consistently used short forms of abbreviations (e.g. HIV Seroconversion) in its synonyms and primary names. We expanded the short forms using our previously developed abbreviations lexicon and added them back to the dictionary.

In order to increase the recall of our normalization module, we have employed the query expansion technique. Query expansion refers to the process of reconstructing a given query often by modifying the terms in a query. The most widely used method is to replace a given term in a term with its synonyms (49). In this study, we expanded a query by appending with disease-related terms such as ‘disorder, syndrome, injury, infection, abnormality’, only when there was no match found in the MEDIC vocabulary. For example, assume that our DNER recognizes ‘posterior reversible encephalopathy’ as an entity. With exact string match setting, no concept is found from the MEDIC vocabulary. With query expansion the identified entity becomes—‘posterior reversible encephalopathy syndrome’ matching with MEDIC synonym for concept identifier ‘D054038’ whose primary name is—‘Posterior Leukoencephalopathy Syndrome’. This type of query expansion assists in overcoming rigid exact match where concept mapping has failed due to a missing term.

**Figure 5.**
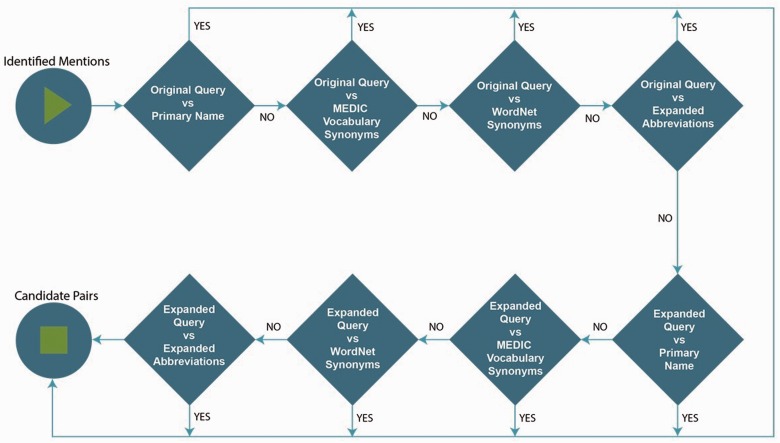
Representation of the developed priority rules.

*Priority rules*. Though exact string matching based dictionary lookup was rigid and strict, we were faced with multiple exact matches for a given query in the dictionary because same names were found as synonyms under multiple MeSH concept IDs and the expansion of dictionaries and queries created duplication of concept names across multiple MeSH concept IDs. Thus, in order to overcome this issue, we implemented few priority rules to determine the final normalized ID. The priority rules were developed to counter attack the negative effects of enhanced dictionary and query expansion, which are logically represented in Figure 5. The highest priority is given for an exact match between the original query, i.e. entity recognized by our DNER module and the primary name in MEDIC. The least priority is given to a match between expanded query and expanded abbreviation entries in MEDIC. The priority rules implemented to some extent limited the number of candidate pairs. Few more rules were required to nominate the final MeSH ID from all available candidate pairs. In situations where there were more than one exact matches, the entries retrieved were checked for the frequency of MeSH concept ID in retrieved records and the MeSH ID with highest frequency is nominated as final candidate. If the frequency was tied, by default the first entry was selected. In any other situations the recognized entity was assigned ‘−1’ representing no MeSH concept ID available to match.

## Results

The dataset from BioCreative V CDR Track (9) was used in this study to develop the proposed methods. The dataset included 1500 PubMed abstracts broken down into training, development and test sets. Both training and development sets were used to train the CRF model. Disease mentions in the dataset were assigned with MeSH IDs using the MEDIC vocabulary. The standard metrics precision (P), recall (R) and F-measure (F) were used to evaluate the performance of the developed modules by assessing true positives (TPs), false positives (FPs) and false negatives (FNs). For evaluating the performance of DNER and DNORM, the outputs of the developed modules for a given article are compared with the set of diseases within the document annotated by human annotators. Table 1 illustrates the distribution and characteristics of the dataset.

**Table 1.** Summary distribution and characteristics of the training, development and test set

	Training set	Development set	Test set
No. of documents	500	500	500
No. of sentences^a^	4597	4604	4800
No. of tokens	108 378	107 668	113 290
Average word count	216.76	215.34	226.58
No. of disease mentions	4182	4244	4424
No. of MeSH IDs (excluding disease mentions without any IDs)	4252	4328	4430
No. of disease mentions without MeSH IDs	32	16	61
No. of unique disease mentions	1384	1254	1337
No. of unique MeSH IDs	664	604	645

^a^Sentence and token stats are generated using Stanford PTBTokenizer.

### DNER performance comparison

We compared our DNER module with BANNER (13) in Table 2 to see how its performance compared to similar DNER tool. BANNER was trained using the default, IOB tagging format and features. In comparison to our DNER module, BANNER demonstrated less precision. We also noticed that the PTBTokenizer used in our DNER module tokenizes more accurately than BANNER’s tokenizer.

**Table 2.** Comparison of DNER module performance

Run	NER Performance at mention-level					
	TP	FP	FN	P	R	F
BANNER	3348	854	1076	0.80	0.76	0.78
Our DNER module	3351	637	1073	**0.84**	0.76	0.80
Our DNER module + Post-processing	3529	811	895	0.81	**0.80**	**0.81**

The bold value signifies highest value with in the column.

### Baseline methods

We implemented four dictionary lookup baseline methods to compare the performance of the proposed methods. In the first baseline method, we tried to find an exact string match for each primary name in MEDIC to a term or phrase in each document of the test. Thus, we refer to this baseline method as the simple dictionary lookup because it resembles the first type of the dictionary-based disease identification method introduced in the ‘Related work’ section. For the rest, the three tools—BeCAS, MetaMap and OBA—discussed earlier were used. They are all based on dictionary lookup and have been widely used as baseline systems (20, 25, 27) to compare various types of normalization approaches (12, 50). For BeCAS, the BeCAS REST web service was used to process the BioCreative V CDR dataset. Each abstract from the dataset was sent to the BeCAS server to annotate disorders. The server returned with identified disease entities and their corresponding UMLS CUIs. The UMLS CUIs were used to obtain corresponding MeSH IDs. For MetaMap, the parameters and configuration were optimized for best performance. MetaMap was restricted to MeSH terminology as source under a relaxed data model. We also used MetaMap’s term processing, word sense disambiguation, ignored word order and allowed only unique abbreviation variants. MetaMap web application programming interface (API) which interacts with MetaMap server in batch was used. Finally, the OBA baseline method was implemented using REST web service. The OBA was configured to find concepts from MeSH terminology. MetaMap and OBA were restricted to find concepts only from disease related semantic types presented in Appendix 2.

### DNORM performance

Table 3 illustrates the performance of all baseline methods. OBA outperformed other baseline methods and BeCAS had poor performance. It is interesting to find that BeCAS which uses deterministic finite automatons, performed worse than simple dictionary lookup. However, BeCAS has shown similar type of performance in previous studies (51, 52).

**Table 3.** Baseline methods performance on the test set

Baseline method	DNORM					
	TP	FP	FN	P	R	F
Simple dictionary lookup	1341	1799	647	0.43	**0.67**	0.52
BeCAS	413	197	1575	**0.68**	0.21	0.32
MetaMap	1272	950	716	0.57	0.64	0.60
OBA	1219	592	772	0.67	0.61	**0.64**

The bold value signifies highest value with in the column.

The performance of the developed method on the test set are presented in Table 4. The first configuration is very similar to the baseline method simple dictionary lookup, except the mentions are recognized by our CRF model and exact string matched with primary entries in the MEDIC vocabulary, instead of direct string matching on the documents. The remaining configurations included improvements like abbreviation resolution, enhanced dictionaries, query expansion and priority rules. Two configurations yielded an F-measure of 0.77. It is interesting to notice that query expansion and priority rules improved the F-measure from 0.73 to 0.77. The F-measure remains 0.77 after adding the DNER post-processing. Overall, the post-processing helped in increasing TPs, however it also led to more FPs.

**Table 4.** Performance of proposed methods on the test set

Configuration#	Configuration description	DNORM					
		TP	FP	FN	P	R	F
1	DNER + Dictionary lookup	758	65	1230	0.92	0.38	0.54
2	1 + Abbreviation resolution	760	65	1228	0.92	0.38	0.54
3	2 + MEDIC vocabulary synonyms	1177	105	811	0.92	0.59	0.72
4	3 + WordNet synonyms	1220	121	768	0.91	0.61	0.73
5	4 + Query expansion + Priority Rules	1342	158	646	0.89	0.68	0.77
6	5 + NER post-processing	1371	184	617	0.88	0.69	0.77

### Execution speed

Table 5 presents the response times taken by the baseline methods and our best configuration to process the test set for five runs using web APIs. BeCAS, OBA and our proposed methods are exposed via REST web services API, where as MetaMap’s web API is not REST-based but allows to interact with MetMap web-based scheduler using batch processing. Shorter response times allow to scale the process to larger collection of documents. Though our proposed methods achieved shortest response time among all baseline methods’ web APIs, it is important to note that the performance of these APIs depend on the infrastructure used to host the API servers, utility load and number of concurrent users. Unfortunately, this information on baseline systems is not available. Our REST server used an Ubuntu machine with 8GB memory powered with Intel® Core™ i7-3770, 3.40 GHz × 8 processor and one concurrent user connected. Nevertheless, the results presented here are very useful in planning experiments and resources for researchers who want to use these publicly available systems on larger collection of documents.

**Table 5.** Processing speed (in seconds per document) for publicly available DNORM systems on the test set

Run	MetaMap	OBA	BeCAS	Configuration 5
1	1.03	12.98	0.62	0.31
2	1.14	12.81	0.51	0.32
3	1.01	13.09	0.51	0.3
4	1.04	13.08	0.45	0.3
5	1.21	12.75	0.46	0.3
**Average** **response** **time (s)**	1.09	12.94	0.51	**0.3**

The bold value signifies highest value with in the column.

## Discussion

Our experiment results demonstrate that the performance of dictionary lookup based DNORM can be improved by combining with the proposed enhanced dictionary and query expansion techniques. Here, we compared our DNORM module performance with another normalization tool which is based on pairwise learning to rank method (pLTR) (12). The pLTR method (TP: 1370, FP: 179, FN: 618) achieved an F-measure of 0.77 on the test set for DNORM, which is the same with that of our configuration 5. From the results, it is evindent that dictionary lookup using exact string matching does provide competetive results in automatic identification of diseases with few minor additional improvements. Furthermore, our method had less execution time when tested locally on the same machine. The pLTR method locally took an average of 3 min to process the test set (500 documents with an average word count of 226.58), whereas our method took 2 min 10 s. The above results suggest that the dictionary lookup DNROM can be easy to extend; with right combination of techniques it can achieve competitive results and has fast execution speed and highly scalable.

### Error analysis

An error analysis was manually performed to identify the possible causes of FNs and FPs in DNORM and at the same time understand why a few additional techniques have not performed well. We chose 200 FPs (30%) and FNs (70%) together randomly from our best configuration and started analyzing those errors. A number of issues were observed; majority of the errors were related to dictinary enhancement (18%), exact string matching (26%) and entity recognition (38%).

*Errors caused by dictionary enhancement.* Dictionary enhancement is an important technique for DNORM, but it also introduced additional issues. The use of WordNet to generate synonyms resulted in wrong and duplicate entries. One of the WordNet synonym for ‘Azotemia (MESH ID: D053099)’ is ‘Uraemia’ which is also WordNet synonym for ‘Uremia (MESH ID: D014511)’. Another issue is errors in dictionary entries. For example, consider the query ‘Colon Cancer’, our system yields two candidate pairs with MeSH IDs D003110 and D015179 where both concepts included ‘Colon Cancer’ as MEDIC synonyms but D015179 actually refers to ‘colorectal neoplasms’. Thus, fixing these type of entries manually and enhancing the dictionary is required.

*Errors caused by matching methods.* The major issue with exact strict matching is that it follows very strict rules and sometimes fails to map entities which are varying slightly and not represented in the vocabulary. For example, our approach assigned ‘−1’ for ‘chronic hepatitis C virus infection’ entity. However, there is an entry for ‘hepatitis C virus infection’ in the MEDIC vocabulary. One-way to overcome this challenge is to employ matching methods like phrase and term matching, which are proximity based matchings and more relaxed compared to exact string matching. We implemented the above two proximity based matching (more details can be found in Appendix 3) and integrated them into our system. The results are presented in Table 6. Interestingly, the improvement in the R metric did not reflect in the P metric, which further went down. This is mainly due to the priority rules, which failed to nominate the right candidate from obtained candidate pairs because of the situation when the frequency of MeSH IDs observed in candidate pairs is a tie. We believe that these issues can be overcome by employing similarity scores or additional priority rules. Finally, the current scoring method was only based on the matching between query terms with MEDIC primary name and synonyms. Embedding definition and broad categories information into the similarity score calculation would further improve the results.

**Table 6.** Performance of proposed methods on test set

Configuration	Norm					
	TP	FP	FN	P	R	F
Configuration 5 + Term match	1444	777	544	0.65	0.73	0.69
Configuration 5 + Phrase match	1419	339	569	0.81	0.71	0.76

*Errors caused by entity recognition.* Overall our DNER module perform better than BANNER, however several issues were noticed. For example, our DNER module repeatedly recognized ‘APC’ (adenomatous polyposis coli) as a disease entity but sometimes failed to recognize mentions which are abbreviated like PPH (pulmonary hypertension) and AIN (interstitial nephritis). Our abbreviation resolution also failed in identifying the above two abbreviations. Employing abbreviation specific features similar to gazetteer features using abbreviation lexicon would have assisted CRF model to recognize these abbreviated mentions more effectively (44). Our DNER module tends to recognize mentions in long form. For instance, ‘Rhabdomyolysis in a hepatitis C virus infected’ was recognized as one entity instead of recognizing ‘Rhabdomyolysis’ and ‘hepatitis C virus infected’ as two separate entities. Another issue is that our module failed to recognize overlapping mentions such as ‘AMI/GI bleeding’, which includes AMI—Acute Myocardial Infarction (MeSH ID: D009203) and Gastro Intestinal bleeding (MeSH ID: D006471). Our DNER recognized ‘bleeding’ as a disease name and as a result was mapped to D006470 referring to bleeding as a general term while D006471 refers to a more specific Gastrointestinal bleeding. Finally, the post-processing step improved our performance mainly by reducing FNs and increasing TPs. However, it also sometimes increased FPs. For example, if our DNER module recognized the FP disease mention ‘plasticity’ once, which is mentioned three times in the abstract, the post-procssing ended up increasing the FP counts by three times. Furthermore, when there are no long forms mentioned in documents and an abbreviation was identified by our abbreviation finder or DNER module, expanding long form based on short form frequency in abbreviation lexicon is not robust. For instance, ‘secondary pulmonary hypertension (SPH)’ was incorrectly assigned to ‘Spherocytosis, Type 1’ (MeSH ID: C567159) by our system because spherocytosis can also be abbreviated as SPH. We also explored using the abbreviation resolution algorithm developed by Schwartz and Hearst (48) directly, instead of our abbreviation resolver but it showed similar results. Therefore, more sophisticated disambiguation techniques should be applied. The above errors caused by DNER and post-processing occupied 28 and 10% respectively against all error types. Therefore, we believe that improving the performance of DNER would improve the performance of DNORM.

## Availability

We have shared our trained models, configuration files, enhanced dictionaries, abbreviation files and expanded queries employed in this study at https://github.com/TCRNBioinformatics/DiseaseExtract. The developed methods are also available for other researchers via web services (REST API) at the same link, together with a simple web application demonstrating our methods.

## Conclusion

In this study, we developed a CRF-based DNER module and dictionary look up method in conjunction with enhanced dictionary and query expansion techniques to normalize disease mentions. Overall, the developed method performed better than several baseline methods. The query expansion improved performance of normalizing entities to MeSH IDs. The exact string matching based dictionary lookup, with right combination of techniques can achieve competitive results. Dictionary lookup based normalization is easy to extend with additional techniques, has fast execution speed and highly scalable. However, further improvements must be made to improve DNORM module performance. For example, one of the major limitations of dictionary lookup based normalization is that it does not consider contextual information. For example, ‘dyskinesia’ can be either mapped to D004409 (Dyskinesia, Drug-Induced) or D020820 (Dyskinesia). Depending on the information in the document, normalization systems should be able to select D004409 as the context of its usage in the text is it was drug induced. Thus, in our future work, we would like to integrate contextual information to expand queries. Also, we would like to focus on improving, (1) the dictionary enhancement technique to avoid duplicates in standard terminologies, (2) proximity based matching for candidate pair generation, (3) priority rules, (4) abbreviation resolution and (5) DNER.

## Appendix 1

### Comparison of dictionary lookup based tools

**Table:**

	MetaMap	cTAKES	OBA	BeCAS
Overall Pipeline	NP → Lexical variants → String matching (Exact & Partial) → Custom score → Disambiguation	Norm → NP → Non-lexical variants → Partial string matching → No disambiguation	Mgrep → String matching (Exact & Partial) → Semantic Expansion>	Modules for PubMed article fetching, Sentence splitting → tokenization → lemmatization → POS tagging → chunking → Partial matching
Dictionary Lookup Matching Type	Partial matching using custom score	Partial matching	Partial matching using rules and semantic expansion	Partial matching using deterministic finite automatons
Abbreviation Resolution	Yes	No	No	Yes
Query Expansion	Lexical variants generated using SPECIALIST lexicon and Lexical Variant Generation (LVG) tools	Non-lexical variants (variations of head & modifiers within noun phrases.)	Semantic expansion (hierarchical and mapping info of ontologies)	Synonyms and Orthographic variants
Dictionary Enhancement	No	Enriched with synonyms from UMLS and a Mayo-maintained list of terms	No	No
Word Sense Disambiguation	Yes	No	Yes	No
Entity Type	All semantic types in UMLS	Disorders/diseases with a separate group for signs/symptoms, test/procedures, anatomy and medication/drugs	All semantic types in UMLS	Species, anatomical concepts, miRNAs, enzymes, chemicals, drugs, diseases, metabolic pathways, cellular components, biological processes, genes, proteins and molecular functions
Terminologies	UMLS	SNOMED and RxNORM	Ontologies listed on NCBO BioPortal	UMLS LexEBI JoChem NCI Metathesaurus miRBase Gene Ontology
Availability	Desktop, Local Java API, Web API, Web Portal	Desktop	REST API, Virtual machine and Web Portal	REST API, Python Command line client and Web Portal

## Appendix 2

### Semantic types used for MetaMap and OBA

**Table:**

UMLS semantic type	UMLS semantic type code	UMLS semantic type acronym
Congenital abnormality	T019	Cgab
Acquired abnormality	T020	Acab
Injury or poisoning	T037	Inpo
Pathologic function	T046	Patf
Disease or syndrome	T047	Dsyn
Mental or behavioral dysfunction	T048	mobd
Cell or molecular dysfunction	T049	comd
Experimental model of disease	T050	Emod
Anatomical abnormality	T190	Anab
Neoplastic process	T191	Neop
Sign or symptom	T184	Sosy

## Appendix 3

### Comparison of exact, phrase and term matches

**Table:**

Exact match	Phrase match	Term match
Each term in the query should be present in the dictionary entry and their order should be strictly maintained. Matching dictionary entry must have only those terms mentioned in query and no additional terms allowed.	Each term in the query should be present in the dictionary entry and their order should be strictly maintained. Dictionary entry may have other terms before or after the query terms.	Each term in the query should be present in the dictionary entry and order is not maintained. Dictionary entry must have at least one query term.
Example: The query ‘TORCH Syndrome’ will return ‘TORCH Syndrome’ dictionary entry.	Example: The query ‘TORCH Syndrome’ will return ‘TORCH Syndrome’ as well as ‘Pseudo-TORCH Syndrome’ entries. For example, the query ‘TORCH Syndrome’ will return ‘TORCH Syndrome’ as well as ‘Pseudo-TORCH Syndrome’ entries.	Example: The query ‘TORCH syndrome’ will return ‘TORCH syndrome’ as well as ‘Pseudo-TORCH Syndrome’, ‘TORCH’ and ‘Syndrome’ entries. For example, the query ‘TORCH syndrome’ will return ‘TORCH syndrome’ as well as ‘Pseudo-TORCH Syndrome’, ‘TORCH’ and ‘Syndrome’ entries.
